# Reduced free ubiquitin levels and proteasome activity in cultured neurons and brain tissues treated with amyloid beta aggregates

**DOI:** 10.1186/s13041-020-00632-2

**Published:** 2020-06-08

**Authors:** Chul-Woo Park, Byung-Kwon Jung, Kwon-Yul Ryu

**Affiliations:** Department of Life Science, University of Seoul, 163 Seoulsiripdae-ro, Dongdaemun-gu, Seoul, 02504 Republic of Korea

**Keywords:** Ubiquitin, Amyloid beta, Primary neuron, Mouse brain slice, Proteasome activity

## Abstract

Neurodegenerative diseases are characterized by progressive cognitive decline and the loss of neurons in the central nervous system; many are also characterized by abnormal aggregation of misfolded proteins. Ubiquitin (Ub) is a eukaryotic protein that plays pivotal roles in protein degradation and cellular signaling. Ubiquitinated aggregates are observed in neurodegenerative diseases; this ultimately results in reduced levels of available or free Ub. However, it remains unclear whether neurotoxicity arises from the aggregates or a deficiency of free Ub. To investigate this, we treated primary neurons of mouse embryonic brains with amyloid beta (Aβ) 42 and found that free Ub levels were decreased and cell viability was reduced in Aβ42-treated neurons. As reduced levels of free Ub are closely related to impaired function of the proteasome, we evaluated proteasome activity and found that proteasome activity was reduced upon treatment of primary neurons and mouse brain slices with Aβ42. Therefore, we conclude that proteotoxic stress from Aβ42 treatment reduced the levels of available Ub and decreased proteasome activity, resulting in inflammatory stress and apoptosis of neurons.

## Main text

Ubiquitin (Ub) is a highly conserved 76 amino acid eukaryotic protein that plays pivotal roles in proteolysis and cellular signaling [1]. Ub is conjugated via the E1, E2, and E3 enzyme cascades and conjugated Ub is recycled by deubiquitinase (DUB) [2, 3]. The ubiquitin proteasome system (UPS) targets numerous cellular proteins for degradation. However, in many cases, aggregated and disease-specific proteins that are characteristic of certain disorders inhibit the activity of the UPS [4]. The accumulation of Ub conjugates and/or inclusion bodies associated with Ub, the proteasome, and certain disease-specific proteins have been previously reported in a broad array of chronic neurodegenerative diseases. These include the neurofibrillary tangles of Alzheimer’s disease; brainstem Lewy bodies; Bunina bodies in amyotrophic lateral sclerosis; and nuclear inclusions in CAG repeat (polyglutamine) expansion disorders, such as Huntington’s disease, spinocerebellar ataxias, and spinal and bulbar muscular atrophy (Kennedy’s disease) [5–9]. These abnormal deposits of aggregates may induce the depletion of available or free Ub. Although polyubiquitin genes are upregulated in response to proteotoxic stress, if the increase in aggregates exceeds the increase in Ub levels, these deposits of Ub conjugates in the aggregates can disrupt Ub homeostasis. Moreover, reduced levels of free Ub and disrupted Ub homeostasis decrease the capacity of cells to protect against stress conditions [10]. Ataxic mice have a spontaneous recessive mutation that results in reduced levels of the DUB, Usp14, and a free Ub pool that is reduced by approximately 35% in most tissues [11]. However, studies on the correlation between proteotoxic stress and reduced levels of free Ub are lacking.

**Fig. 1:**
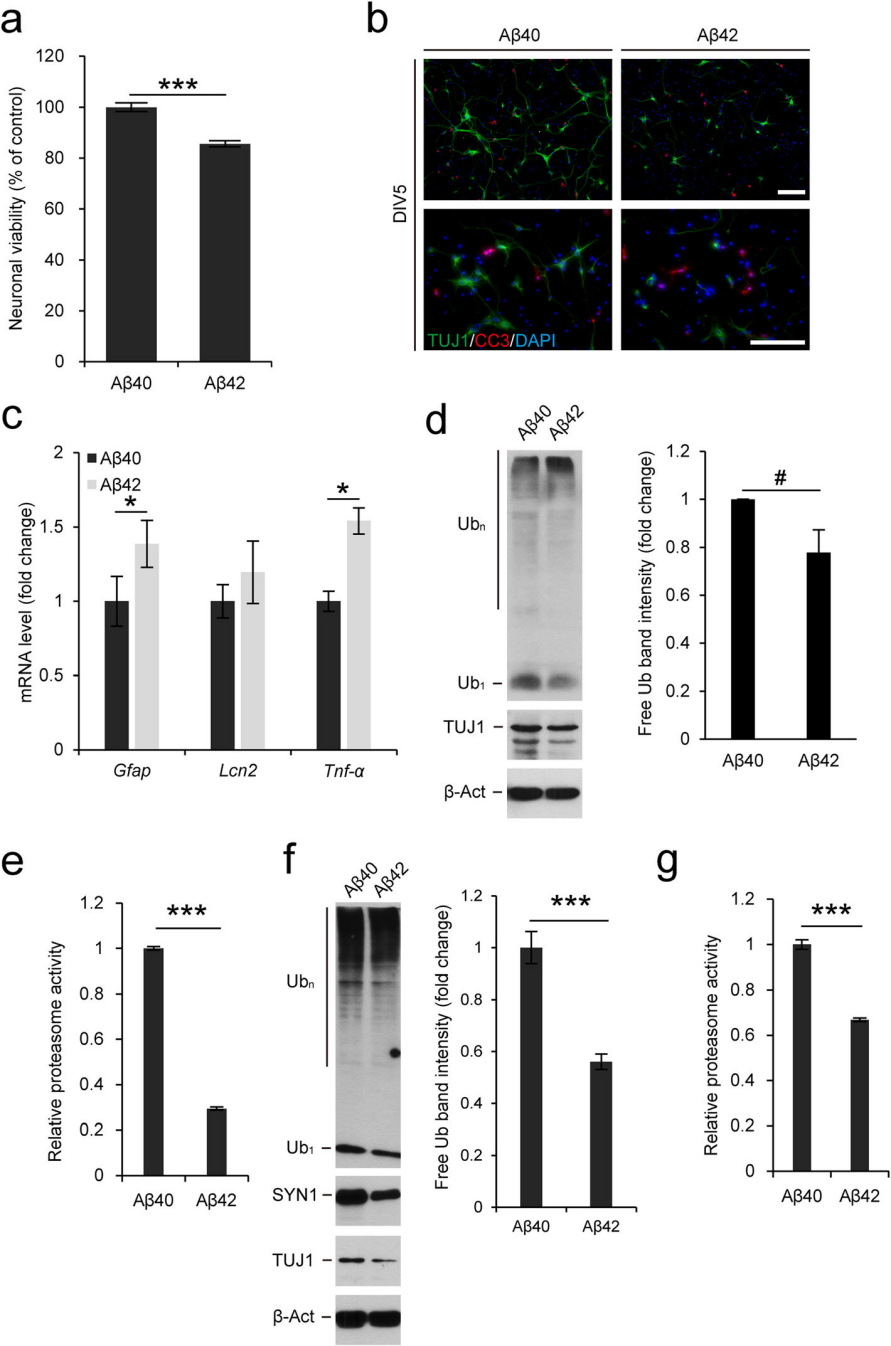
Reduced free Ub levels and proteasome activity in neurons and brain slices treated with Aβ. (a) Cell viability detection with MTT assay. Primary neurons were isolated from mouse embryonic brains at 14.5 dpc and treated with Aβ40 or Aβ42 at DIV1 and analyzed by MTT assay at DIV5 (*n* = 3 per group). (b) Primary neurons were treated with Aβ40 or Aβ42 at DIV1 and stained for TUJ1 and CC3 at DIV5. DNA was visualized with DAPI. Scale bar, 100 μm (c) *Gfap*, *Lcn2*, and *Tnf-α* mRNA levels were determined by qRT-PCR in Aβ40- or Aβ42-treated primary neurons (*n* = 3 per group). The expression levels of *Gfap*, *Lcn2*, and *Tnf-α* were normalized to *Gapdh* levels and are expressed as the fold change relative to Aβ40-treated neurons. (d) Immunoblot detection of Ub (free, Ub_1_; conjugates, Ub_n_) and TUJ1 in Aβ-treated primary neurons. The neurons were treated with Aβ40 or Aβ42 at DIV1 and harvested at DIV5. TUJ1 was used as a neuronal marker and β-actin (β-Act) was used as a loading control (left). Band intensities of free Ub were calculated using ImageJ software, normalized to the loading control β-actin, and are expressed as the fold change relative to Aβ40-treated neurons (*n* = 3 per group) (right). (e) Aβ40- and Aβ42-treated primary neurons were harvested at DIV5 and subjected to a proteasome activity assay (*n* = 3 per group). Proteasome activities were calculated by subtracting the values of MG-132 treated samples from those of experimental samples and are expressed as the fold change relative to Aβ40-treated neurons. (f) Immunoblot detection of Ub, TUJ1, and SYN1 in Aβ-treated brain slice cultures. The slices were treated with Aβ40 or Aβ42 at DEV1 and harvested at DEV5. TUJ1 and SYN1 were used as neuronal markers and β-actin was used as a loading control (left). Band intensities of free Ub were calculated as above (*n* = 4 per group) (right). (g) Aβ40- and Aβ42-treated brain slices were subjected to a proteasome activity assay at DEV5 as above (*n* = 3 per group). All data are expressed as the means ± SEM from the indicated number of samples and representative images or immunoblot results are shown. ^#^*P* < 0.1; ^*^P < 0.05; ^***^P < 0.001 vs control

To investigate the relationship between Ub homeostasis and proteotoxic stress, we used amyloid beta (Aβ) 42 as a proteotoxic stress inducer in primary neurons. It is well known that Aβ42 is prone to assemble into insoluble extracellular fibrils and that neurons uptake these Aβ fibrils to form insoluble intracellular aggregates. To investigate whether Aβ affects cell viability, we pretreated the Aβ42 peptide to induce fibril structure formation [12] and used Aβ40 as a control. Primary neurons at days in vitro 1 (DIV1) were treated with Aβ peptides to evaluate their effects on neural differentiation and development. Using MTT assays, we found that neuronal viability was significantly decreased after Aβ42 fibril treatment (Fig. 1a). Although Aβ42 treatment reduced neuronal viability, it is possible that neuronal structures such as neurites and soma were not affected in the surviving neurons. We checked the morphology of Aβ-treated neurons via immunofluorescence and found that most neurons were damaged upon Aβ42 treatment. Moreover, levels of the apoptosis marker, cleaved caspase-3 (CC3) [13], were increased in Aβ42-treated neurons (Fig. 1b). In our primary neuron cultures, neural stem cells (NSCs) accounted for 50% of the total seeded cell population. Astrocytes can differentiate from NSCs under stress conditions. To investigate whether the differentiation into astrocytes was increased upon Aβ treatment, we determined the expression levels of *Gfap* and found that they were significantly increased in Aβ42-treated neurons (Fig. 1c). To determine whether increased expression levels of *Gfap* induce the reactive astrocyte phenotype, we measured the expression levels of *Lcn2* and *Tnf-α* (Fig. 1c). Lcn2 has previously been reported to be secreted by reactive astrocytes and to induce the apoptosis of damaged neurons [14]. Thus, increased *Lcn2* levels is one of the markers of reactive astrocytes. *Tnf-α* levels were measured to detect the induction of inflammation. Interestingly, there were no differences in *Lcn2* expression levels between Aβ40- and Aβ42-treated neurons, indicating that Aβ42-induced neuronal death was not caused by reactive astrocytes, at least under our experimental conditions. However, neuronal death resulted in increased levels of the inflammatory cytokine, *Tnf-α*.

Reduced free Ub levels are known to affect neuronal viability and in an ataxic mouse model, free Ub levels are decreased in the brain. Therefore, we measured Ub levels in Aβ-treated neurons (Fig. 1d). Aβ42-treated neurons had lower levels of free Ub and higher levels of high molecular weight Ub conjugates, indicating that extracellular Aβ42 peptides induced the formation of intracellular aggregates, with free Ub depletion, resulting in higher levels of neuronal apoptosis than those induced by Aβ40. In fact, expression levels of both polyubiquitin genes, *Ubb* and *Ubc*, and total Ub levels were not different between Aβ40- and Aβ42-treated neurons (Supplementary Fig. 1a), which also supported that reduced levels of free Ub, not total Ub, affected cell viability. To determine the correlation between reduced levels of free Ub, Aβ42, and neuronal viability, we assessed proteasome activity in Aβ42-treated cells, because it was previously reported that Aβ fibrils inhibit proteasome activity, resulting in abnormal protein turnover and neuronal death [15]. Proteasome activity was significantly reduced in Aβ42-treated primary neurons (Fig. 1e). These results suggest that Aβ42 fibrils induced the depletion of available Ub and inhibited proteasome activity, which affected neuronal viability.

Since we observed reduced levels of free Ub and decreased proteasome activity in Aβ42-treated primary neurons, we investigated whether these results could be recapitulated in brain tissue. We first used *Ubb* knockout (KO) mouse brains to confirm whether fluctuated Ub pools affect proteasome activity and found that the proteasome activity was significantly reduced in KO brains (Supplementary Fig. 1b). To determine the effect Aβ42 on brain tissue, we sectioned mouse brains and cultured the brain slices with Aβ42. In Aβ42-treated brain slices cultured ex vivo for 5 days (DEV5), we observed reduced levels of free Ub and the neuronal markers, TUJ1 and SYN1 (Fig. 1f). We further examined proteasome activity to determine whether free Ub levels also correlated with proteasome activity in Aβ42-treated brain slices. Proteasome activity in brain slices was also significantly decreased at DEV5 (Fig. 1g).

In conclusion, we demonstrated that reduced free Ub levels may be a marker of proteotoxic stress after Aβ42 treatment. We found that free Ub levels, proteasome activity, and neuronal viability were well correlated under neuronal- and brain-specific Aβ-induced proteotoxic stress. We suggest that Aβ aggregates reduce free Ub levels and disrupt Ub homeostasis, which may play a key role in decreasing proteasome activity and inducing neuronal apoptosis. Therefore, the regulation of free Ub levels may be a novel therapeutic strategy for various neurodegenerative diseases.

## Supplementary information

**Additional file 1.** Supplementary information accompanies this paper at http://doi.org/.

## Data Availability

All data analyzed in this study were included in this article. Materials and methods are presented in the supplementary information.

## Abbreviations

Aβ40: Amyloid beta 40
Aβ42: Amyloid beta 42
DEV: Days ex vivo
DIV: Days in vitro
Ub: Ubiquitin
