# Supplementary material for: Reduced free ubiquitin levels and proteasome activity in cultured neurons and brain tissues treated with amyloid beta aggregates
Source: Mol Brain. 2020 Jun 8;13:89. doi: 10.1186/s13041-020-00632-2 (PMC7281939; doi:10.1186/s13041-020-00632-2)
Supplement: Supplementary file 1 — Additional file 1. Supplementary information accompanies this paper at http://doi.org/. [file 13041_2020_632_MOESM1_ESM.docx]

**Supplementary information**

**Reduced free ubiquitin levels and proteasome activity in cultured neurons and brain tissues treated with amyloid beta aggregates**

Chul-Woo Park, Byung-Kwon Jung, Kwon-Yul Ryu^*^

*Department of Life Science, University of Seoul, Seoul 02504, Republic of Korea*

^*^Corresponding author: Kwon-Yul Ryu, Department of Life Science, University of Seoul, 163 Seoulsiripdae-ro, Dongdaemun-gu, Seoul 02504, Republic of Korea

*E-mail address:* kyryu@uos.ac.kr (K. -Y. Ryu).

**Materials and methods**

**Mouse studies**

All mice were kept in plastic cages with ad libitum access to food and water. All experimental protocols including breeding, euthanasia, and dissection of embryonic brains, were approved by the University of Seoul Institutional Animal Care and Use Committee (UOS IACUC; approval no. UOS-170517-1). All animal procedures were performed in accordance with the relevant guidelines and regulations approved by the UOS IACUC.

**Primary neuronal culture**

At 14.5 days post coitum (dpc), embryonic brains were dissected and the cerebellum and meninges were removed in Hank’s balanced salt solution (HBSS) without calcium and magnesium. Processed brains were transferred to Accutase (Sigma) with DNase I, triturated using a 1,000 μL pipette tip, and incubated for 15 min at 37°C. An equal volume of neuronal growth medium, consisting of Neurobasal medium supplemented with B-27 supplement (Invitrogen), 1× GlutaMax (Gibco), and 1% antibiotics/antimycotics (Cellgro), was then added. After centrifugation, the pellet was resuspended in neuronal growth medium and strained through a 40 μm nylon mesh. The resulting cells were then centrifuged again and cultured in the same medium, on a cell culture dish coated with poly-D-lysine (Sigma) and laminin (Invitrogen).

**Amyloid beta (**Aβ) **preparation**

Synthetic Aβ40 and Aβ42 peptides (AnaSpec) were dissolved in 100% HFIP (Sigma) to a concentration of 1 mM. The dissolved solution was evaporated in a fume hood and the dry peptide film was stored at -20°C until use. To form aggregates, the peptides were first resuspended in DMSO to a concentration of 5 mM, after which 10 mM HCl was added to bring the peptides to a final concentration of 100 μM. The peptide solution was then incubated for 24 h at 37°C.

**Proteasome activity assay**

Harvested cells or tissues were homogenized in lysis buffer (50 mM NaH_2_PO_4_ [pH 7.5], 100 mM NaCl, 10% glycerol, 5 mM MgCl_2_, 0.5% NP-40, 5 mM ATP, and 1 mM DTT) with 1 mM PMSF, 1 μg/μL aprotinin, and 1 μg/μL leupeptin as protease inhibitors. Lysates were centrifuged at 13,000 rpm for 15 min at 4°C and the resulting supernatant was removed to measure protein concentration using the Bradford protein assay (Pierce). To measure chymotrypsin-like activity, the fluorogenic substrate, Suc-LLVY-AMC (Bioclone), was added to the lysates (10 μg) and assays were performed at 37°C in a proteasome assay buffer (50 mM Tris-HCl [pH 7.5], 1 mM EDTA, 1 mg/mL BSA, 1 mM ATP, and 1 mM DTT). As a control, MG-132 (10 μM) was added to samples before performing assays. Proteasome activities were determined based on the intensity of free AMC generated from the cleavage of the substrate and the values of MG-132 treated samples were subtracted from those of experimental samples. Fluorescence was measured using a fluorescence plate reader (SpectraMax M2e; Molecular Devices) at excitation and emission wavelengths of 380 nm and 460 nm, respectively. An emission cutoff filter of 435 nm was used.

**Cell viability assay**

To perform an MTT assay, medium was removed and replaced with an MTT working solution (500 mg/mL). Cells were then incubated for 4 h at 37°C. The converted dye was solubilized with DMSO and the absorbance of the converted dye was measured at 550 nm, with background subtraction at 650 nm.

**Immunoblot analysis**

Cell pellets or tissue samples were lysed in RIPA buffer (25 mM Tris-HCl [pH 7.6], 150 mM NaCl, 1% NP-40, 1% sodium deoxycholate, and 0.1% SDS) with 1 mM PMSF, 1 μg/μL aprotinin, and 1 μg/μL leupeptin as protease inhibitors and incubated on ice for 30 min. Lysates were centrifuged at 13,000 rpm for 10 min at 4°C and the supernatant was removed to measure protein concentration using a BCA protein assay (Thermo Fisher Scientific). To detect total Ub levels, samples were treated with Usp2cc as previously described [1]. Cell or tissue lysates (15 μg) were subjected to SDS-PAGE, followed by immunoblot detection using anti-Ub (Santa Cruz Biotechnology), anti-SYN1 (Millipore), anti-TUJ1 (Santa Cruz Biotechnology), or anti-β-actin (Santa Cruz Biotechnology) antibodies. Horseradish peroxidase (HRP)-conjugated goat anti-mouse IgG was used as the secondary antibody (Enzo Life Sciences).

**Immunofluorescence analysis**

Cells grown on poly-D-lysine-coated coverslips were fixed in 4% paraformaldehyde for 10 min at room temperature (RT), permeabilized with 0.3% Triton X-100/PBS, and blocked with 3% BSA/PBS for 1 h at RT. Fixed cells were incubated with anti-TUJ1 (Santa Cruz Biotechnology), anti-GFAP (Millipore), or anti-cleaved caspase-3 (Millipore) antibodies in blocking solution, at 4°C overnight. They were then washed with PBS and incubated with Alexa Fluor 488- or 555-conjugated goat anti-mouse or donkey anti-rabbit IgG (Invitrogen), with DAPI, for 1 h at RT. Cells were then mounted using Prolong Gold antifade reagent (Invitrogen). Immunofluorescence images were visualized with an Axio Imager A2 microscope (Car Zeiss).

**Quantitative reverse transcription PCR (qRT-PCR)**

Total RNA was isolated from cultured cells using the TRI Reagent (Molecular Research Center) and 1 μg of total RNA was reverse transcribed. The resulting cDNA samples were used as templates for PCR. Before reverse transcription, samples were treated with DNase I (Invitrogen) for 15 min at RT to eliminate DNA contamination and DNase I was then inactivated by treatment with EDTA for 10 min at 65°C. Reverse transcription was performed using SuperiorScript II Reverse Transcriptase (Enzynomics), according to the manufacturer's protocol. qRT-PCR was performed using a SYBR qPCR 2x Master Mix (Enzynomics) and an iCycler system, with iCycler iQ5 software version 2.0 (Bio-Rad). mRNA expression levels were normalized to the levels of *Gapdh*. The primers used for qRT-PCR were as follows: *Gfap*-F (5′-CGA GTC CCT AGA GCG GCA AAT G-3′); *Gfap*-R (5′-GTA GGT GGC GAT CTC GAT GTC-3′); *Lcn2*-F (5′-CTG AAT GGG TGG TGA GTG TG-3′); *Lcn2*-R (5′-GCT CTC TGG CAA CAG GAA AG-3′); *Tnf-α*-F (5′-TCT CAT CAG TTC TAT GGC CC R-3′); *Tnf-α*-R (5′-GGG AGT AGA CAA GGT ACA AC-3′); *Ubb*-F (5′-TCT GAG GGG TGG CTA TTA A-3′); *Ubb*-R (5′-TGC TTA CCA TGC AAC AAA AC-3′); *Ubc*-F (5′-GTT ACC ACC AAG AAG GTC-3′); *Ubc*-R (5′-GGG AAT GCA AGA ACT TTA TTC-3′); *Gapdh*-F (5′-GGC ATT GCT CTC AAT GAC AA-3′); and *Gapdh*-R (5′-CTT GCT CAG TGT CCT TGC TG-3′)

**Brain slice culture**

 Mice were sacrificed at postnatal day 5 and immediately decapitated under sterile conditions. Brains were rapidly removed and glued (Loctite) to the specimen plate of a vibratome (Leica) and trimmed with a commercial razor blade in chilled dissection medium (HBSS with calcium and magnesium [Gibco], with 6 mg/mL glucose and 1% antibiotics/antimycotics). Serial coronal sections of the hippocampal region were cut at a thickness of 200 μm and collected in fresh dissection medium. Three organotypic slices were carefully transferred to a 0.4 μm membrane insert (SPL) in a 6-well plate. Slices were incubated with culture medium, consisting of Neurobasal A medium supplemented with B27 supplement, 1× GlutaMax, and 1% antibiotics/antimycotics, in a humidified 5% CO_2_ atmosphere at 37°C. The entire medium was replaced with fresh medium on the following day.

**Statistical analysis**

All experimental results are presented as means ± SEM. Data were analyzed by Student’s t-test, and differences were considered statistically significant if *P* < 0.1.

**Supplementary Figure**

**
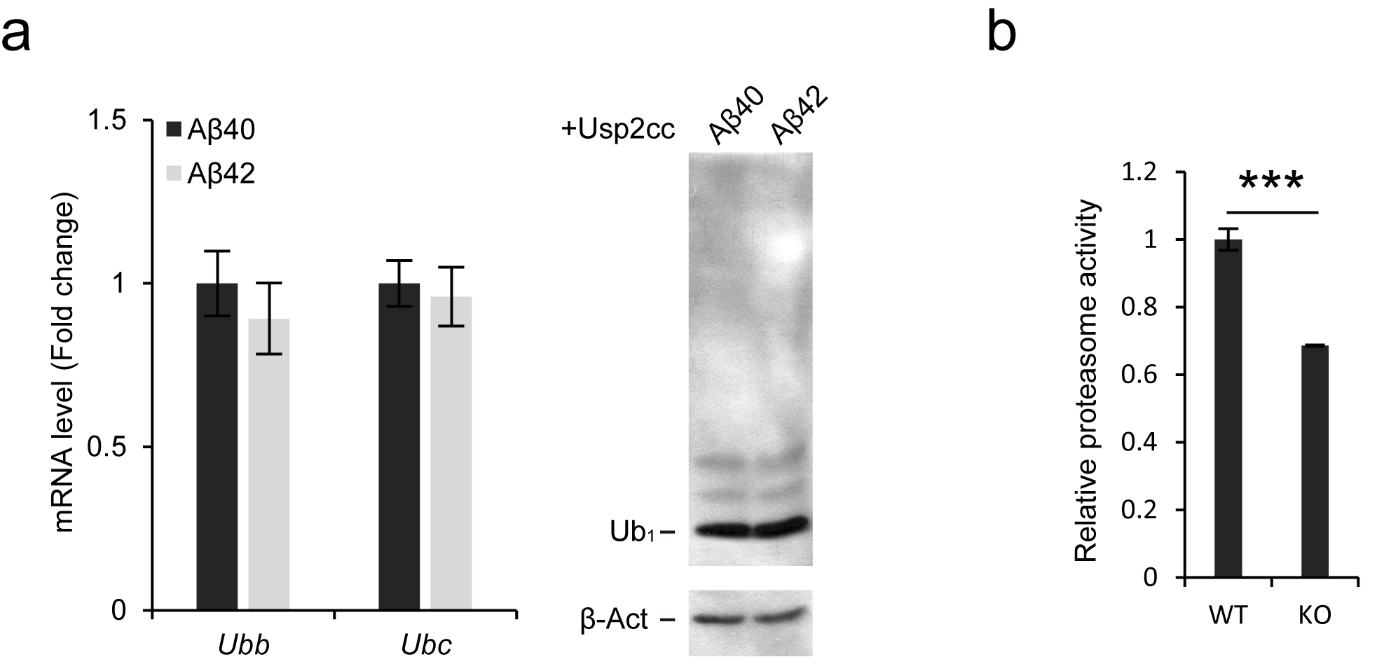
**

**Supplementary Fig. 1** Total Ub levels in Aβ-treated neurons and proteasome activity under Ub pool fluctuation. (a) Investigation of total Ub levels by qRT-PCR and immunoblot analysis. *Ubb* and *Ubc* mRNA levels were determined by qRT-PCR in Aβ40- or Aβ42-treated primary neurons (n = 3 per group) (left). The expression levels of *Ubb* and *Ubc* were normalized to *Gapdh* levels and are expressed as the fold change relative to Aβ40-treated neurons. Total Ub levels were determined in Aβ40- or Aβ42-treated primary neurons after conversion of all Ub conjugates to free Ub monomer (Ub_1_) via Usp2cc treatment (right). (b) Reduced proteasome activity in *Ubb* knockout (KO) mouse brains. The hypothalamic regions from wild-type (WT) and *Ubb* KO mice were isolated at postnatal day 20 and subjected to a proteasome activity assay (n = 3 per group). Proteasome activities were calculated by subtracting the values of MG-132 treated samples from those of experimental samples and are expressed as the fold change relative to WT control. All data are expressed as the means ± SEM from the indicated number of samples. ^***^*P* < 0.001 vs control.

**Supplementary Reference**

1. Ryu KY, Baker RT, Kopito RR. Ubiquitin-specific protease 2 as a tool for quantification of total ubiquitin levels in biological specimens. Anal Biochem. 2006; 353(1):153-5.
